# Supplementary material for: Exopolysaccharide from Lactobacillus plantarum HY7714 Protects against Skin Aging through Skin–Gut Axis Communication
Source: Molecules. 2021 Mar 16;26(6):1651. doi: 10.3390/molecules26061651 (PMC8002305; doi:10.3390/molecules26061651)

molecules

MDPI

Article

Exopolysaccharide from *Lactobacillus plantarum* HY7714 protects against skin aging through skin–gut axis communication

Kippeum Lee †, Hyeon Ji Kim, Soo A kim, Soo-Dong Park, Jae-Jung Shim and Jung-Lyoul Lee *

[1] R&BD Center, Korea Yakult Co Ltd., 17086, 22, Giheungdanji-ro 24 beon-gil, Giheung-gu, Gyeonggi-do, Republic of Korea; joy4917@hanmail.net; hyeonjk@re.yakult.co.kr (H.J.K.); freebie@re.yakult.co.kr (S.A.K.); soodpark@re.yakult.co.kr (S.-D.P.); jjshim@re.yakult.co.kr (J.-J.S.); jlleesk@re.yakult.co.kr (J.-L.L)

† This author is first author

* Correspondence: jlleesk@re.yakult.co.kr

Figure S1. Culture of EPS-producing lactic acid bacteria. Cultured strains were grown in MRS broth. (A) *L. plantarum* HY7714. (B) *L. plantarum* HY7711.

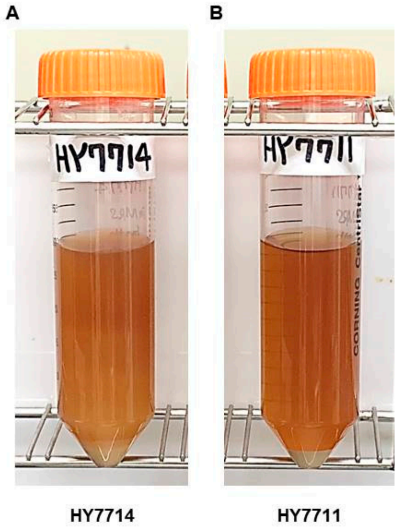

Figure S2. High-performance liquid chromatography (HPLC) chromatogram after acid hydrolysis of EPS produced by *L. plantarum* HY7714 and HY7711. (A) Acid-hydrolyzed products of EPS from *L. plantarum* HY7714, *L. plantarum* HY7711, and standards (ribose, fructose, mannose, glucose, sucrose, and maltose).

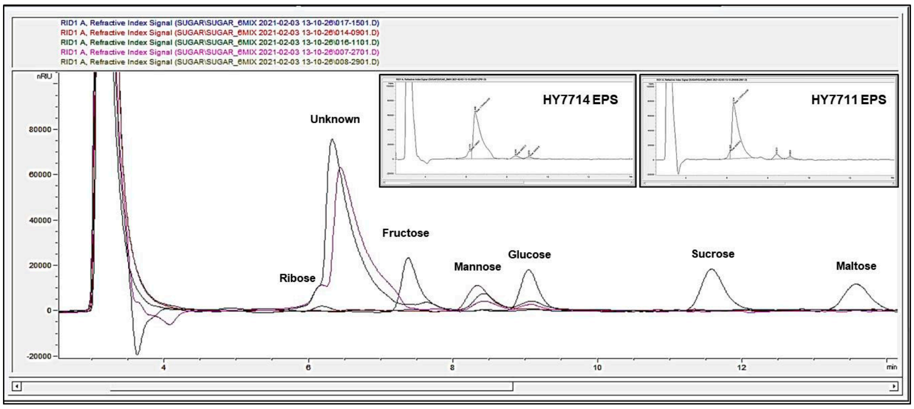

Supplement: Supplementary file 1 [file molecules-26-01651-s001.pdf]
